# Bayesian additive regression trees for machine learning to classify benign vs atypical lipomatous tumors on MRI

**DOI:** 10.1093/radadv/umaf036

**Published:** 2025-10-06

**Authors:** Felipe Godinez, Nimu Yuan, Rijul Garg, Yasser G Abdelhafez, Anik Roy, Hande Nalbant, Cyrus P Bateni, Jinyi Qi, Michelle Zhang, Sonia Lee, Ahmed W Moawad, Khaled M Elsayes, Michele Guindani, Lorenzo Nardo

**Affiliations:** Department of Radiology, University of California, Davis, Sacramento, CA 95817, United States; University of California Davis Comprehensive Cancer Center, Sacramento, CA 95817, United States; Department of Biomedical Engineering, University of California, Davis, Davis, CA 95616, United States; Department of Electrical Engineering and Computer Sciences, University of California, Berkeley, Berkeley, CA 94720, United States; Department of Radiology, University of California, Davis, Sacramento, CA 95817, United States; Department of Radiotherapy and Nuclear Medicine, South Egypt Cancer Institute, Assiut University, Assiut, 71515, Egypt; Statistics, Indian Statistical Institute, Kolkata, 700108, India; Department of Radiology, University of California, Davis, Sacramento, CA 95817, United States; Department of Radiology, University of California, Davis, Sacramento, CA 95817, United States; Department of Biomedical Engineering, University of California, Davis, Davis, CA 95616, United States; Department of Diagnostic Radiology, McGill University Health Center, Montreal, QC H3A0G4, Canada; Department of Radiological Sciences, University of California, Irvine, Irvine, CA 92697, United States; Department of Diagnostic Radiology, Mercy Catholic Medical Center, Darby, PA 19023, United States; Department of Diagnostic Imaging, University of Texas MD Anderson Cancer Center, Houston, TX 77030, United States; Department of Diagnostic Imaging, University of Texas MD Anderson Cancer Center, Houston, TX 77030, United States; Department of Biostatistics, University of California, Los Angeles, Los Angeles, CA 90095, United States; Department of Radiology, University of California, Davis, Sacramento, CA 95817, United States; University of California Davis Comprehensive Cancer Center, Sacramento, CA 95817, United States

**Keywords:** machine learning, Bayesian additive regression trees, radiomics, atypical lipomatous tumors, MRI, tumor classification

## Abstract

**Background:**

Atypical lipomatous tumors (ALTs) are aggressive fat cell tumors that are distinguished from benign lipomas (SL) mainly through histopathology. Biopsy is needed for suspicious cases but can miss malignancy, so complete surgical removal and examination are essential. MRI is used but often can't differentiate ALT from SL. We introduce a machine learning method for tumor classification.

**Purpose:**

To characterize the classification performance of a Bayesian additive regression trees (BART) model, built from MR radiomic features, and compare it to the readings of a musculoskeletal radiologist in classifying atypical lipomatous tumors (ALTs) from simple lipomas.

**Materials and Methods:**

Retrospective data were collected from 5 medical institutions in North America, for a total of 437 patients; the mean age was 58 years ±12 years, with 248 men and 287 women. At each institution, at least T1-MRI images without contrast were collected from patients with suspected ALT prior to surgery. Histopathology was used as the reference standard. Radiomic features extracted from the MRI images were used to train the BART model and a random forest model for comparison of classification performance using a 10-fold cross-validation. Both models were compared with the classifications of an experienced (>10 years) musculoskeletal radiologist who scored the images on a 5-point scale.

**Results:**

A cohort of 423 patients was included, and 1132 radiomic features were extracted from each MR study. The BART model had an accuracy, sensitivity, and specificity of 77.07% (72.76%-80.99%), 77.67% (71.36%-83.16%), and 76.50% (70.28%-81.97%), respectively, when utilizing all predictors and aggregating training and testing data from all the cohorts, approximating the human reader at 78.72% (74.51%-82.53%), 76.21% (69.80%-81.85%), and 81.11% (75.25%-86.09%), respectively. In the external validation, the average area under the curve (AUC) value across cohorts between the BART model and the human reader differed by 0.04 AUC points. From the receiver operating characteristic curve, the AUC was calculated to be 84.72% (81.00%-88.50%) and 84.74% (81.00%-88.50%) for the BART and human reader, respectively.

**Conclusion:**

This study demonstrated that the BART model can distinguish ALT from lipoma with diagnostic performance comparable to an experienced human observer.

**Abbreviations** 

ALT = Atypical lipomatous tumors; AUC = Area under the curve; BART = Bayesian additive regression trees; SL = Simple lipoma; GBM = Gradient boosting regression; gray level co-occurrence matrix (GLCM), GLRM = gray level run length matrix;GLSZM = gray level size zone matrix, GLDM = gray level dependence matrix; NGTDM = neighborhood gray tone difference matrix HR = Human observer; LASSO = Least absolute shrinkage and selection operator; ML = Machine learning; RF = Random forest; PACS = Picture archiving and communication system; EHR = Electronic health record

**Summary** 

A Bayesian additive regression trees model, built from MRI radiomic features, performed comparably to an experienced radiologist for classifying benign from atypical lipomatous tumors, using pathology as the reference standard.

**Key Results** 

- Bayesian additive regression trees (BART) model, using MRI radiomics, can distinguish pathologically verified atypical lipomatous tumors (ALT) from benign ones.
- BART matched the human reader in diagnostic performance in external validation when batch effects were accounted for.
- BART prediction could be developed as a tool to offer a second opinion, increase diagnostic confidence, and decrease the necessity for biopsy confirmation.

## Introduction

Atypical lipomatous tumors (ALTs) are locally aggressive mesenchymal neoplasms composed of proliferating adipocytic cells, showing abnormal nuclei in stroma and adipocytes.^1^ The distinction between ALT and its benign counterpart, the simple lipoma (SL), is currently based on histopathology.^2^ In the clinical management of SL tumors, with no malignant suspicions, routine observations are sufficient. In the case of a radiographically suspicious tumor, a biopsy is often pursued to evaluate for malignancy. However, needle biopsy does not exclude malignancy due to the possibility of sampling error. For suspicious lesions, complete surgical removal and pathology are needed for a definitive diagnosis.^3^ MRI is the standard imaging modality for evaluating lipomatous tumors; however, the diagnostic performance for discriminating ALT from SL has been variable, with consistently poor specificity.^4^^,^^5^

Machine learning (ML) has been used for the classification task in various types of cancer.^6–8^ ML models using radiomics, an approach to high-dimensional image-derived data that generates hundreds of image features,^9^ have been employed to differentiate ALTs from lipomas with promising results.^10–13^ In an ALT classification study by Fradet et al,^10^ radiomics data were used to show that ML methods outperformed deep learning methods. Spaanderman et al showed that combining conventional ML models can perform the ALT classification task across multiple medical centers well using images segmented with a deep learning model.^14^

The Bayesian additive regression trees (BART)^15^ ML model is proposed here as a novel approach for ALT classification that uses Bayesian priors to control the behavior of the decision tree. The prior information can be used to regularize training and prevent overfitting data to achieve a more generalizable ML model^16^ by reducing the individual tree size.^13^ Unlike traditional tree-based models like random forest (RF) or gradient boosting, BART provides the benefit of CIs and posterior distributions for predictions and parameters. BART naturally allows for variable selection. For example, by examining how often variables are used in splitting or how much they contribute to predictive accuracy. This helps in feature interpretation and dimensionality reduction. The earliest use of BART in cancer detection is a validity study to predict the presence of colon cancer from microarray colon datasets,^17^ where BART was shown to be more sensitive than traditional ML.

In this work, we report an ALT classification method based on the BART model approach from MRI image radiomic analysis. The aim of this work is to benchmark the classification performance of the BART model in comparison to expert musculoskeletal radiologist readings. To the best of our knowledge, this is the first report on the use of BART for ALT classification.

## Materials and methods

### Patient cohort

In a retrospective study, cohort data were collected with Institutional Review Board approval from the University of California, Davis; the reliance mechanism within the University of California; and individual Institutional Review Board approvals at participating institutions outside the University of California. Sharing of anonymized clinical information and imaging data was regulated by data transfer agreements. Multiple institution data acquired during the years 2008 to 2018 were collected from 5 medical centers in North America for a total of 423 patients (Table 1) that had a full resection of a lipomatous mass. In a previous report, a portion of these data (185 patients) was used to train a deep learning auto-segmentation model.^18^ The inclusion criteria included an MRI performed prior to surgery, full resection of the lipomatous mass, and a pathology report confirming the diagnosis of either ALT or SL. Patients were excluded if they had incomplete imaging studies, poor-quality images, or no pathologic verification. The data underlying this article will be shared on reasonable request to the corresponding author.

**Table 1.** Demographic data for patient cohort with MRI lipomatous tumors confirmed pathologically.

	ALT prevalence (%)	Cohort size			Age		Sex	
		Total	Included	Excluded	Mean	SD	Male	Female
All sites	48.70	437	423 [206]	14	58	12	248	287
Site A	63.32	229	229 [145]	0	59	12	101	128
Site B	29.55	47	44 [13]	3	57	14	31	35
Site C	35.09	58	57 [20]	1	58	13	25	35
Site D	28.38	83	74 [21]	9	58	12	34	45
Site E	36.84	20	19 [7]	1	58	11	7	12

The number of pathological ALT patients is shown in brackets.

Abbreviation: ALT = atypical lipomatous tumor.

### MR imaging protocol

Images included at least axial T1-weighted scans without contrast acquired on 1.5T or 3.0T magnetic field strengths. The protocol included a nonfat-suppressed fast spin echo sequence. Further details on the MRI protocols are described in Nardo et al.^4^ The types of scanners used are tabulated in Table S1.

### Expert human reader review

An experienced musculoskeletal radiologist with more than 10 years of experience reviewed all the acquired MR series for each patient and categorized the impression on a 5-point diagnostic score, where 1 = definitely lipoma, 2 = probably lipoma, 3 = equivocal, 4 = probably ALT, and 5 = definitely ALT. The radiologist was blind to the ground truth from histopathologic analysis. Two threshold approaches were evaluated, with the equivocal category (score 3) considered once as ALT (more sensitive approach) and then with score 3 considered lipoma (more specific approach).

### Bayesian additive regression tree model

In BART, priors or the label probably distribution encode expert beliefs about prediction structures before observing radiomic features, guiding the model toward simpler tree ensembles. These priors moderate confidence by ensuring each tree is a weak learner, preventing overfitting, and accurately reflecting uncertainty in posterior predictions. As a result, BART delivers accurate predictions along with a measure of uncertainty, such as CIs. For a detailed and intuitive description of the BART model, the reader is directed to the study of Tan and Roy^16^ and the Supplementary Information. For comparison, the BART model was matched against a standard RF model.

### Image processing

**Figure 1.**
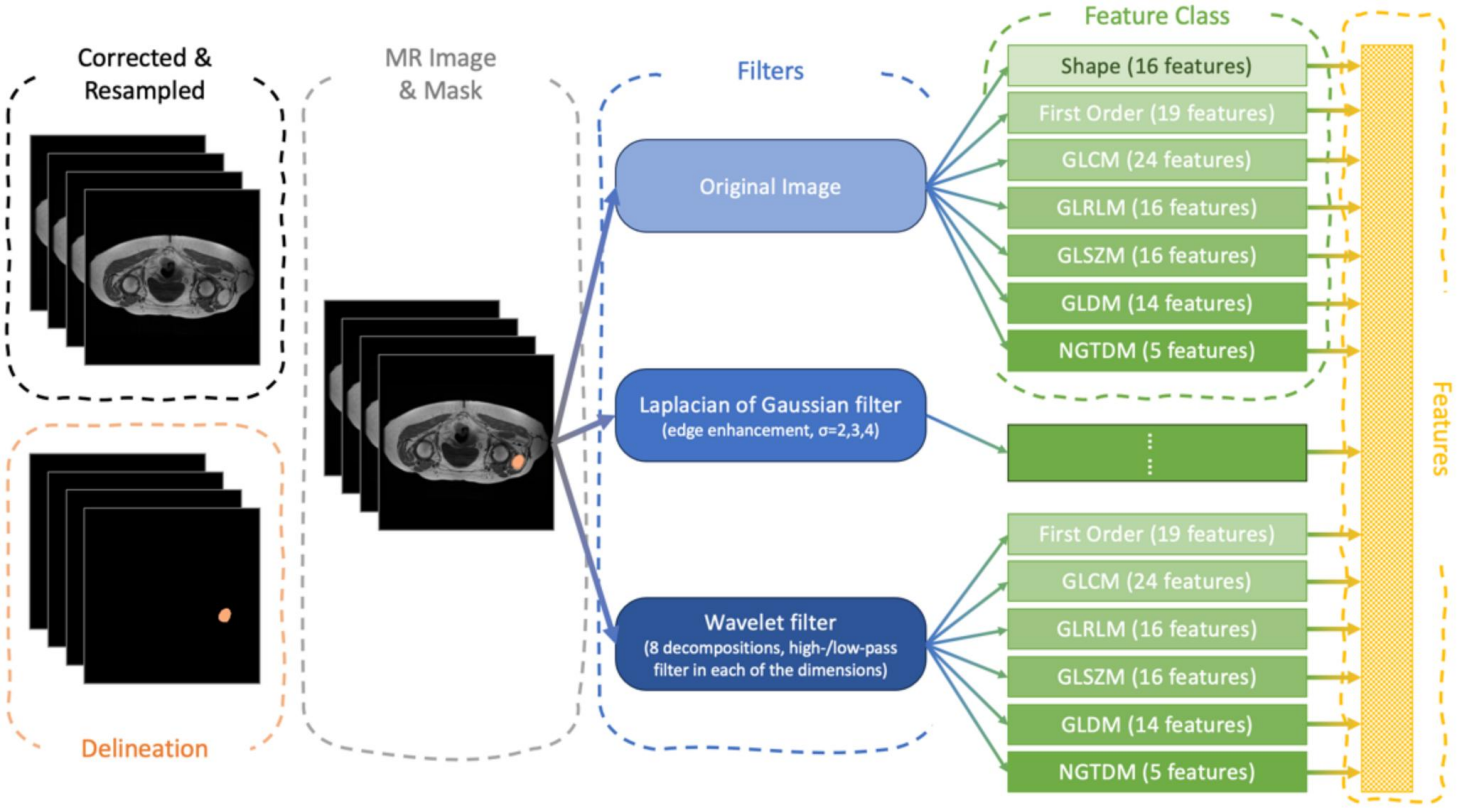
Image processing scheme. MR images were corrected using N4 Bias Field Correction, and the corrected MR images and masks were further resampled to a 2 × 2 × 2 mm^3^ voxel resolution.

The image processing pipeline is shown in Figure 1. The tumor region was delineated in 3D T1w images by manual segmentation methods according to previously described methods^18^ by 1 of 2 readers. Since MR data were acquired from various institutions, scanners, and scanning protocols, the N4 bias field correction algorithm^19^ was applied to correct and normalize the low-frequency intensity non-uniformity present in the MR image across subjects, followed by image resampling to 2.0 mm cubic voxels.

### Feature extraction

The process for MR radiomics feature extraction is rigorously aligned with the image biomarker standardization initiative.^20^ Given that this is a feasibility study, all available Pyradiomics features were selected to create a wide net to capture important image textures. No re-segmentation intensity range was applied, as MR signal intensities are not standardized like Hounsfield units in CT. Gray-level discretization was performed using a fixed bin width of 5 intensity units to ensure consistent texture calculation across patients. In total, 1132 features were computed across the 7 standard PyRadiomics classes: first-order statistics, shape, gray level co-occurrence matrix (GLCM), gray level run length matrix (GLRLM), gray level size zone matrix (GLSZM), gray level dependence matrix (GLDM), and neighborhood gray tone difference matrix (NGTDM)^21^ (Table S2). Features were extracted from both the original images and derived images using different filters, including 3D Laplacian of Gaussian filters (sigma = 2.0, 3.0, 4.0, and 5.0 mm) and 3D wavelet filters (8 different decompositions, each utilizing a combination of high- and low-pass filters across the 3 spatial dimensions). All the features were combined as the input to the BART model. To reduce the impact of institutional biases, feature data were harmonized using the ComBat algorithm.^22^

### Machine learning and training

**Figure 2.**
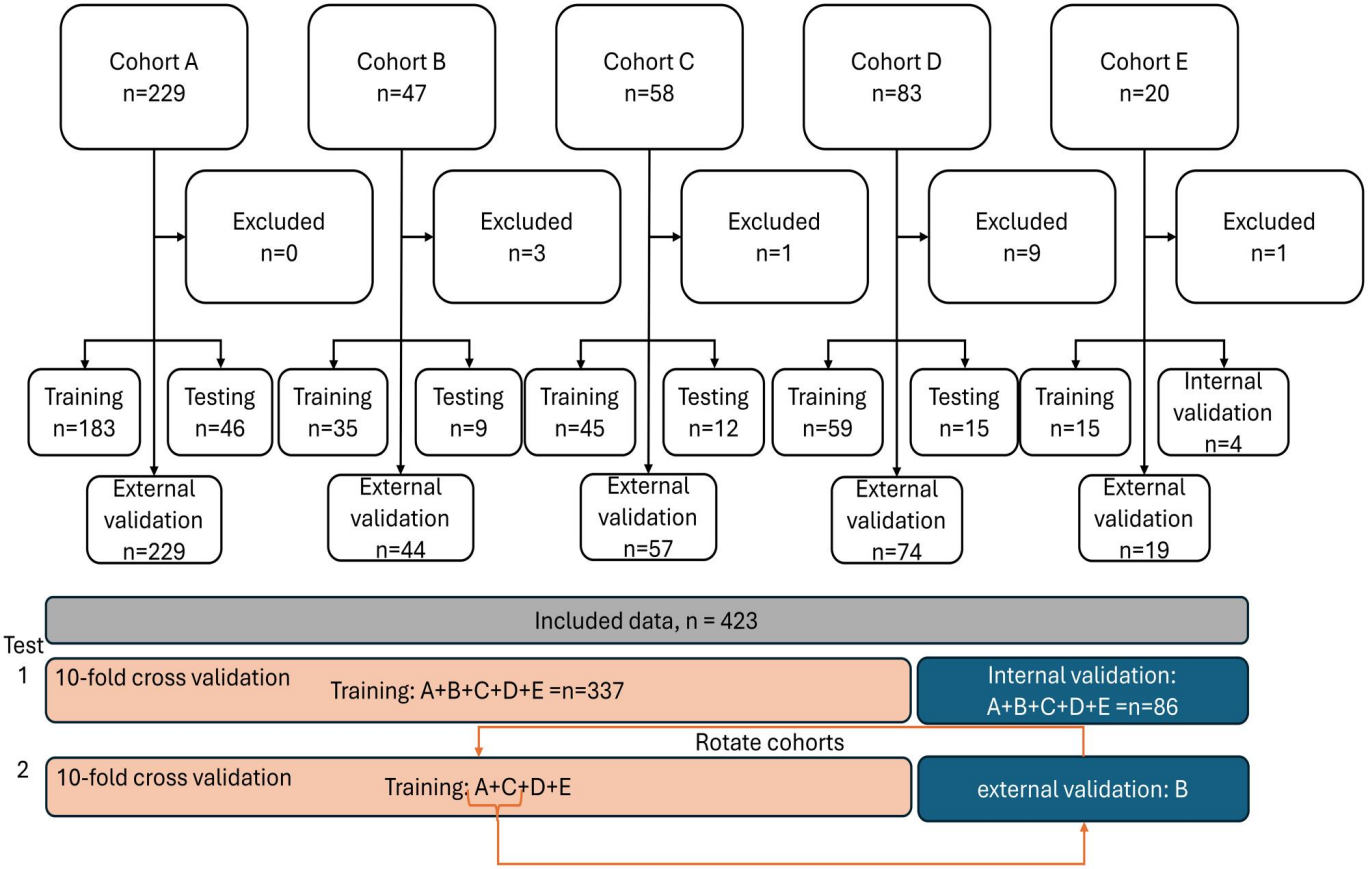
Overview of data flow. Test 1 is the training and testing with all the cohorts included, while test 2 is the external validation, where the model was trained like in test 1, but one cohort was left out for external validation. The external validation was repeated for all cohorts.

The BART model was implemented using the BARTmachine tool,^23^ with 100 trees, 250 burn-in, and 1000 post iterations. The model was trained with an 80%/20% split training/testing using a 10-fold cross-validation, taking 2.9 seconds to build the model (Figure 2). Computations were performed on a MacBook Pro (2021 model) equipped with an Apple M1 Pro chip. The processor features 10 cores and 32 GB of unified memory. Parallelization was used only at the level of replicated simulations, with each dataset analysis executed on a single core, reducing the overall runtime by distributing independent replications across cores. The cross-validation function from the BARTmachine package was used to optimize the BART model by treating Bayesian prior hyperparameters (variance prior, terminal node prior, number of trees) as operational parameters to be tuned via cross-validation.^15^ We wanted to find the optimal performing model according to the decision threshold, as this can be selected arbitrarily by the user. Using the above approach, this optimization was performed for a set (0.32-0.50) of decision thresholds applied to the prediction probability. The decision threshold that best matched the human reader's performance was kept.

External validation was conducted by leaving 1 cohort out during training and using it for testing; this was repeated for all cohorts.

In order to select the most important features, we examine the set of predictor variables used for each splitting rule in each tree and compute the variable inclusion proportion (VIP) across all trees. More specifically, we implement the permutation-based approach^24^ that generates a null distribution for the VIPs and automatically determines the most appropriate threshold to select a predictor. Using BART models, we measured each predictor’s importance via VIP, reflecting how often a predictor appears in tree splits. To account for spurious importance, we created a null distribution by permuting outcomes, refitting the model, and recalculating VIPs. We then assigned a permutation-calibrated *P*-value for each predictor, comparing its real VIP to this null distribution. Small *P*-values suggest the feature’s importance is unlikely due to chance, providing a calibrated measure of predictive contribution. See Tables S3 and S4 for a detailed description and definitions of features and importance scores.

The predictive power of the variable selection was then assessed by training and testing models with the 5, 10, and 15 most important features via 10-fold cross-validation. The RF model was also trained in the same way as the BART model for comparison.

### Random forest classification

The RF model was run on a MacBook Pro M3 with 11 CPU cores and 18 GB RAM. It was trained with 500 trees, importance enabled, and the number of variables set to the square root of the number of features. For the 80/20 batch, the process looped 100 times. Performance metrics were averaged across runs to produce a receiver operating characteristic (ROC) curve. External validation was done as with BART.

### Statistical analysis

The degree of agreement between the BART model and the human reader performance, or the RF model, was assessed with the Cohen’s kappa test. The differences and statistical significance in the proportion of incorrect to correct classifications between the BART model and human reader were evaluated with the nonparametric McNemar’s test.

The ROC curve for the 10-fold cross-validation was generated by calculating the true positive rate and false positive rate for a range of classification probability thresholds between 0.05 and 0.95. The area under the curve (AUC) was calculated using the trapezoid rule, and the DeLongi method was used to compare the BART and human reader ROC, or the BART and the RF model. The BART results were then summarized in a confusion matrix, which provided various performance metrics such as sensitivity, specificity, accuracy, false positive rate, misclassification rate, positive predictive value, and negative predictive value. The 95% CIs were calculated for sensitivity, specificity, AUC, and accuracy using the exact Clopper-Pearson CI method.^25^ For the positive and negative predictive values, a standard logit CI is given by Mercaldo et al^26^; except when the predictive value is 0% or 100%, in which case a Clopper-Pearson CI is reported.

### Model sharing

The ML model and associated code developed in this study are available upon reasonable request for purposes of research and validation, subject to institutional review and compliance with applicable privacy and data protection policies. Interested researchers should contact the corresponding author directly.

## Results

### BART model performance

**Figure 3.**
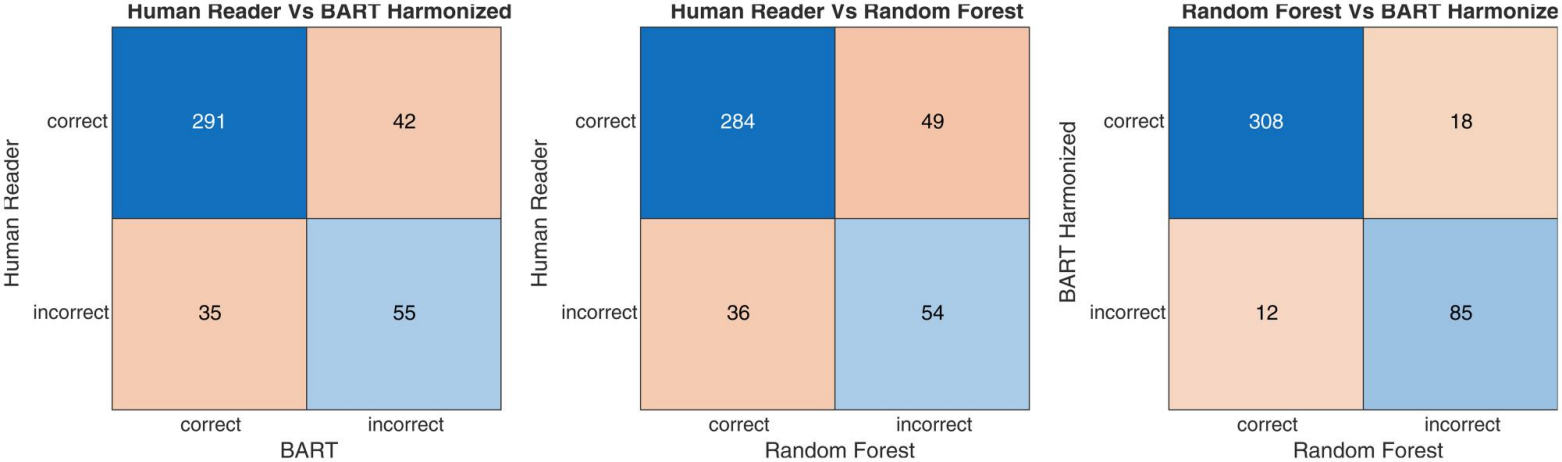
Contingency matrix for human reader and Bayesian additive regression trees (BART) predictions showing the proportions of correct and incorrect predictions (left). The human reader vs the random forest (middle), and BART harmonized vs the random forest (right).

**Figure 4.**
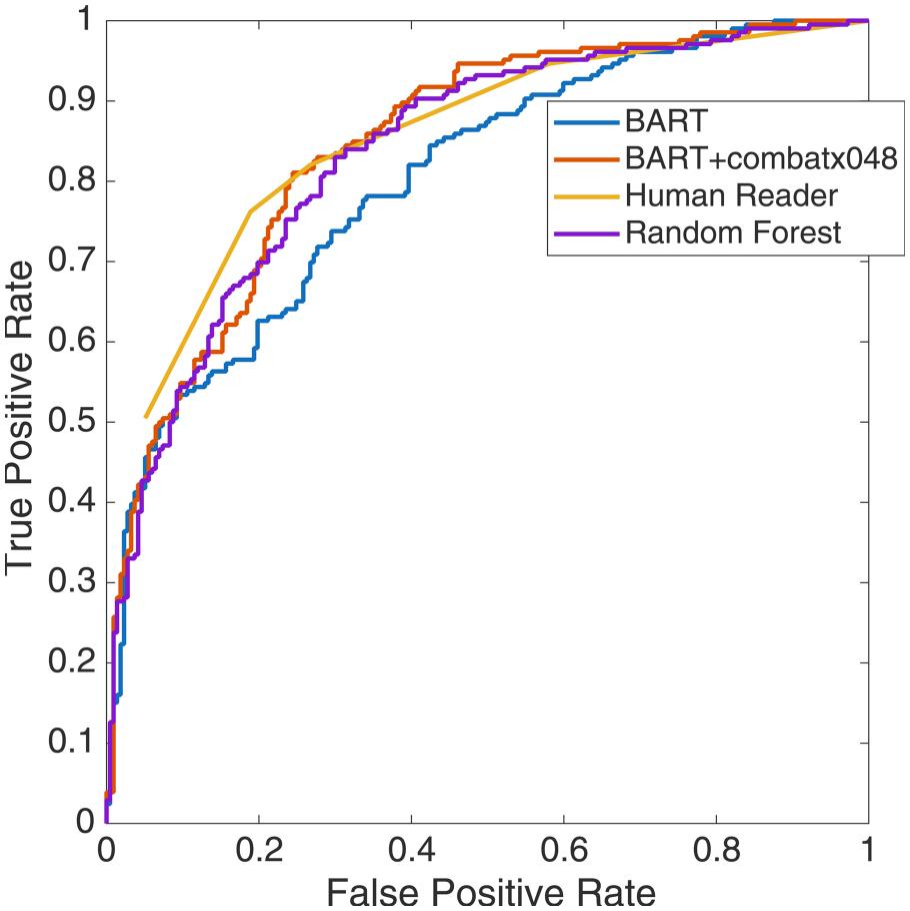
ROC (receiver operating characteristic) curve for the Bayesian additive regression trees (BART) model from the 10-fold cross-validation. The model output was considered for a range of classification probabilities to generate the ROC curve. The area under the curve for the BART with ComBat model and human reader using the 5-point scores was 80.29%, 84.72%, 84.74%, and 83.75%, respectively.

**Figure 5.**
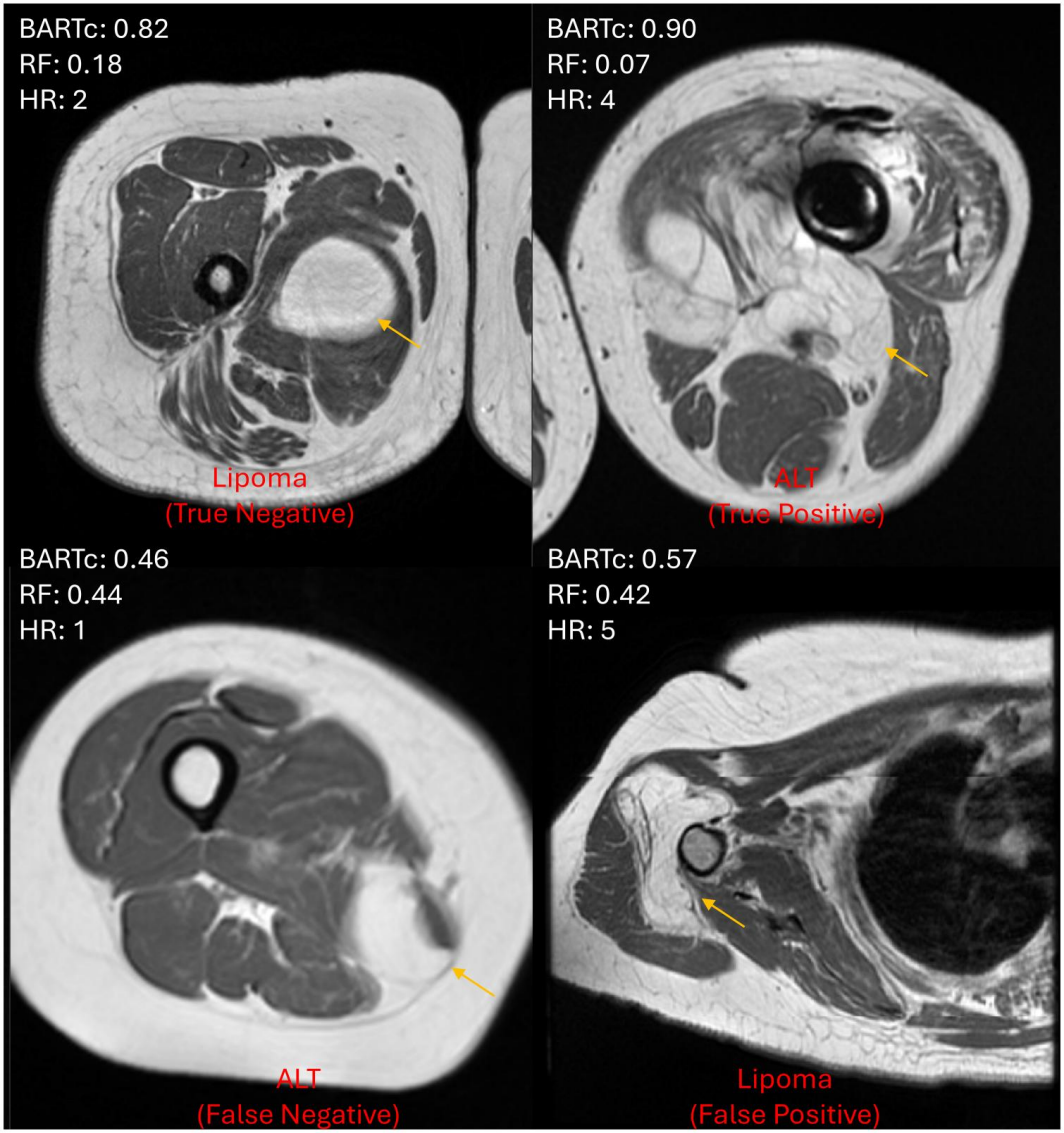
Example images for each case where the human reader (HR) prediction resulted in a false positive/negative or true positive/negative. The BART (Bayesian additive random trees) with combat and RF (random forest) prediction > 0.48 is classified as malignant. The corresponding HR score is shown, where a score >3 is malignant. Where 1 = definitely lipoma, 2 = probably lipoma, 3 = equivocal, 4 = probably atypical lipomatous tumor (ALT), and 5 = definitely ALT (atypical lipomatous tumor).

**Figure 6.**
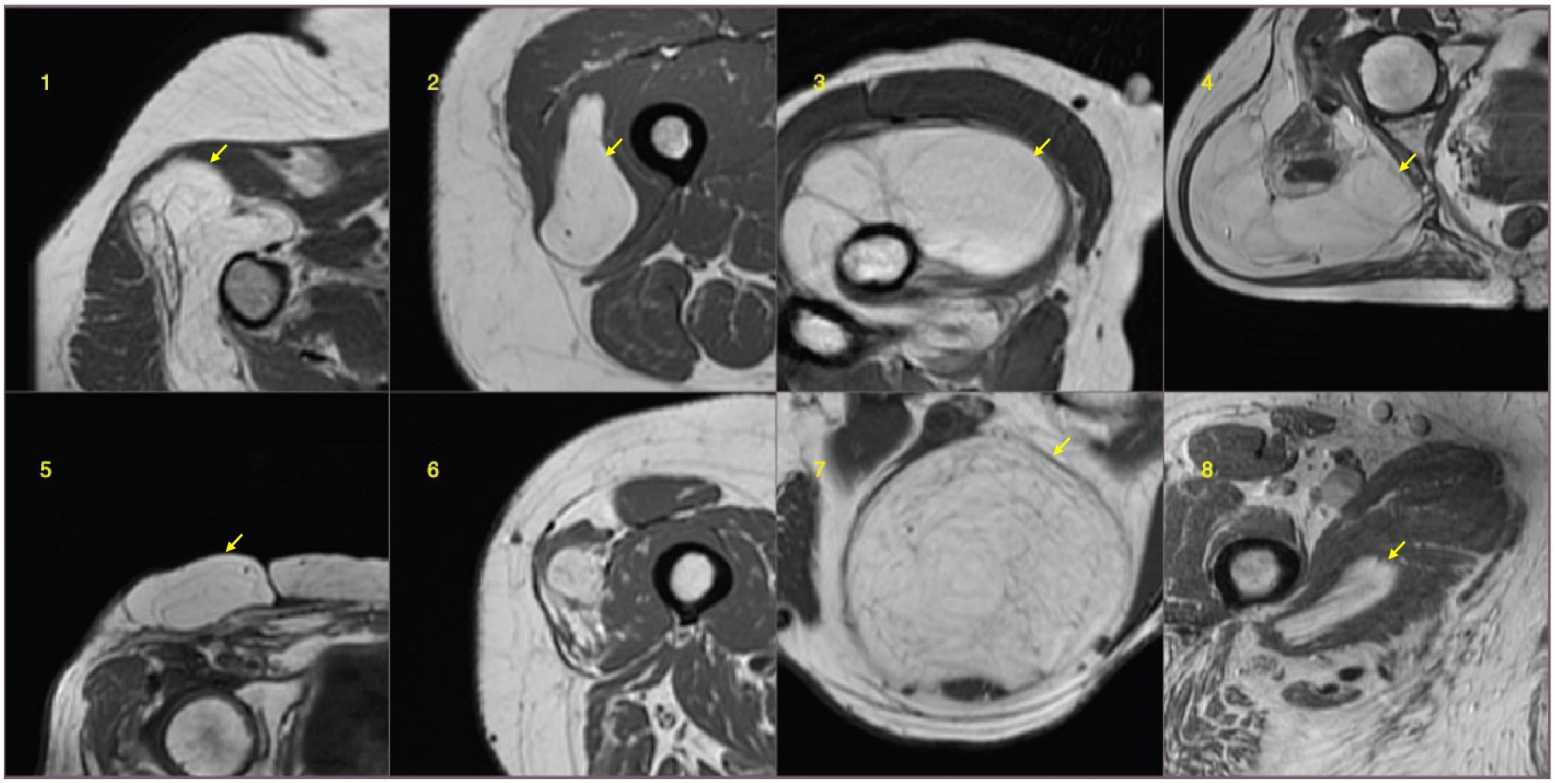
Classification failure examples. The top row images, 1 to 4, are of the false positive type, and the bottom row images, 5 to 8, are of the false negative type for both human reader and BART (Bayesian additive random trees).

**Figure 7.**
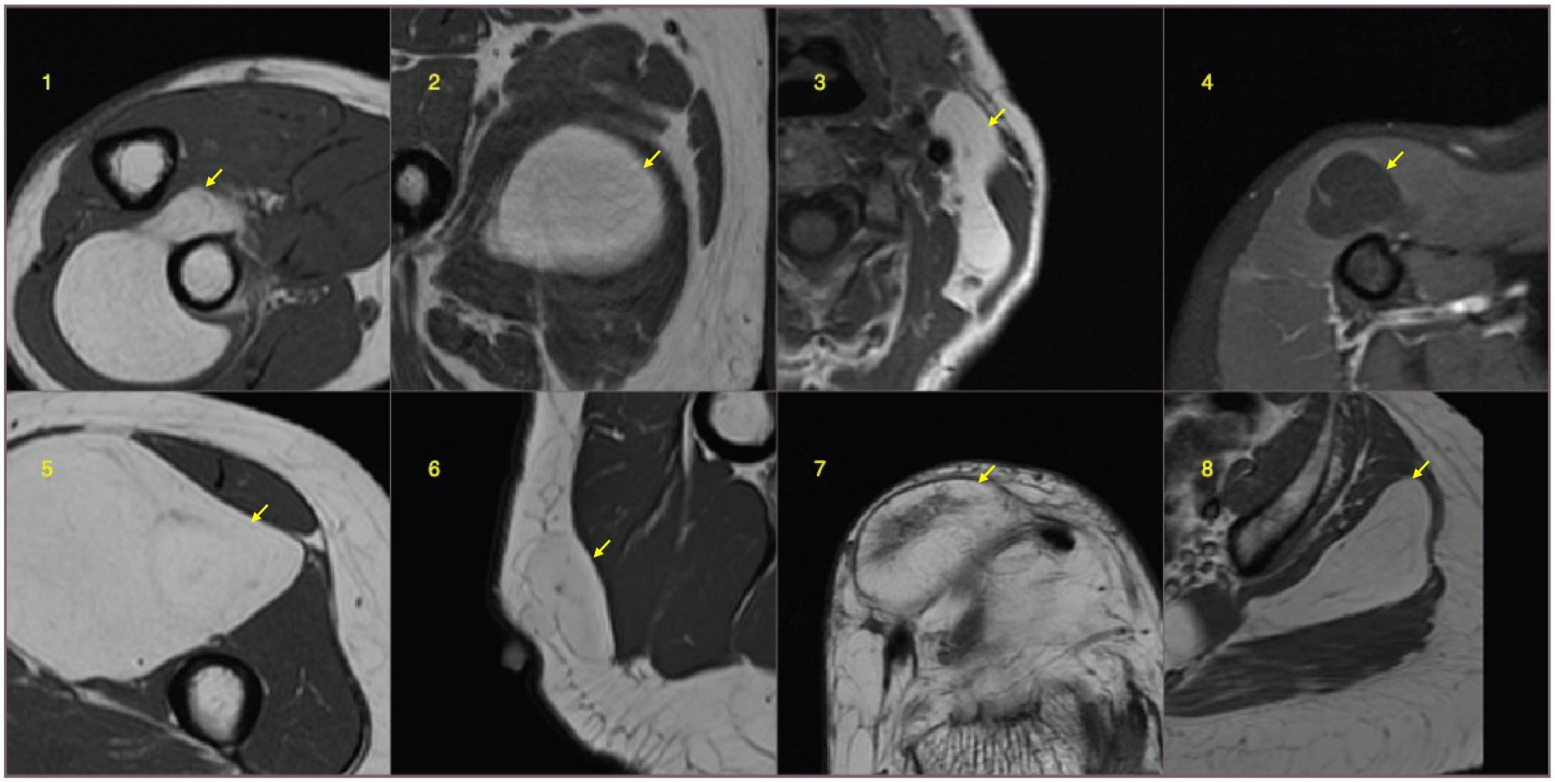
Classification failure examples when BART (Bayesian additive random trees) and the human reader disagree. In the top row images, the BART prediction was a false positive, and human reader prediction was a true negative. In images 1 to 3, the random forest prediction was a true negative. In the bottom row images, the human reader’s prediction was a false positive, and the BART prediction was a true negative.

The confusion matrix for all predictors is shown in Figure S1—the contingency matrix for all predictor combinations is shown in Figure 3. The inter-rater agreement kappa coefficient between predictors is tabulated in Table 2. The kappa was 0.43 between the human reader and the RF model. The difference in proportion of correct predictions/impressions for the human reader and BART model was −1.65% (−0.5.72% to 2.41%) based on the McNemar method; for the human reader and RF, it was −3.31% (−7.89% to 1.28%). The difference was not significant, with *P*-values of .1400 and .1928, respectively. The performance metrics for the 10-fold cross-validation using all predictors are shown in Table 3. The respective AUC values for the BART model without harmonization, BART with harmonization, the RF model, and the human reader with different score thresholds were 80.29, 84.72, 83.75, and 84.74. Note that the ROC performance of the harmonized BART is similar to that of the human reader (Figure 4). Figure 5 shows example images comparing BART and human prediction performance. More example images where both predictors failed are provided in Figures 6 and 7.

**Table 2.** Intra-rater agreement (kappa) and differences in classification performance of lipomatous tumors on MRI between the HR and BART (McNemar).

	BART harmonized vs HR	RF vs HR	BARTComBat vs RF
Kappa inter-rater agreement	0.47 (0.37 to 0.57) SE = 0.051	0.43 (0.33 to 0.53) SE = 0.052	0.80 (0.74 to 0.87) SE = 0.034
McNemar test	−1.65% (−5.72% to 2.41%) *P* = .1400	−3.31% (−7.89% to 1.28%) *P* = .1928	−1.42% (−3.95% to 1.12%) *P* = .3616
AUC comparison *Z*-score/*P*-value	0.00536/.9957	0.359/.7298	0.353/.7238

Abbreviations: AUC = area under the curve; BART = Bayesian additive random trees; HR = human reader; RF = random forest.

Significance at *P* < .05.

**Table 3.** Performance metrics of BART based on the average of 100 replicas, each performing a 10-fold cross-validation, with and without harmonization, RF, and for the expert HR.

	BART	BART harmonized	RF	HR Score >3	HR Score >2
Sensitivity	68.45 (61.62-74.73)	77.67 (71.36-83.16)	76.21 (69.80-81.85)	76.21 (69.80-81.85)	81.55 (75.57-86.60)
Specificity	73.27 (66.86-79.04)	76.50 (70.28-81.97)	75.12 (68.81-80.72)	81.11 (75.25-86.09)	73.27 (66.86-79.04)
Accuracy	70.92 (66.34-75.21)	77.07 (72.76-80.99)	75.65 (71.27-79.67)	78.72 (74.51-82.53)	77.30 (73.01-81.21)
False positive rate	26.75	23.50	24.88	18.89	26.73
Misclassification rate	29.08	22.93	24.35	21.28	22.70
Positive predictive value	70.85 (65.69-75.53)	75.83 (73.46-82.47)	74.41 (69.50-78.76)	79.29 (74.20-83.60)	74.34 (69.72-78.47)
Negative predictive value	70.98 (66.33-75.23)	78.30 (72.76-80.99)	76.89 (72.03-81.12)	78.72 (73.61-82.22)	80.71 (75.64-84.93)
AUC	80.29 (76.07-84.51)	84.72 (80.96-88.48)	83.75 (79.88-87.62)	84.74 (80.98-88.50)	

The 95% CIs are shown in parentheses.

Abbreviations: AUC = area under the curve; BART = Bayesian additive regression trees; HR = human reader; RF = random forest.

### External validation

The AUC values derived from using a single institutional cohort for external validation are tabulated in Table 4. In all external validations, the BART model AUC was not statistically different from the human reader. The average AUC values across cohorts for the human reader, the BART harmonized, and the RF were 0.88, 0.84, and 0.81, respectively. The *Z*-scores are shown in Table 4. The performance, demonstrated as sensitivity, specificity, and accuracy of BART when tested with an external cohort, is shown in Figure S2.

**Table 4.** External validation ROC analysis.

	HR Score >3	BART harmonized	*Z*-score/*P*-value	RF	*Z*-score/*P*-value
Site A	0.79 (0.73-0.85)	0.78 (0.72-0.84)	0.1612/.8719	0.74 (0.68-0.80)	1.051/.2933
Site B	0.83 (0.68-0.98)	0.77 (0.60-0.94)	0.5702/.5685	0.71 (0.53-0.89)	1.0137/.3107
Site C	0.84 (0.72-0.96)	0.79 (0.66-0.92)	0.6300/.5287	0.75 (0.61-0.89)	0.9817/.3262
Site D	0.97 (0.92-1.00)	0.92 (0.84-1.00)	0.9058/.3650	0.91 (0.82-1.00)	1.0074/.3137
Site E	0.97 (0.91-1.00)	0.92 (0.82-1.00)	0.4794/.6316	0.92 (0.82-1.00)	0.5852/.5584
Average AUC	0.88	0.84	–	0.81	–
Difference from HR	–	0.04	–	0.07	–

AUC values for BART are based on the average of 100 replicas, each performing a 10-fold cross-validation with harmonization, and for the HR results, for a single cohort excluded from training for validation testing. The 95% CIs are shown in parentheses.

Abbreviations: AUC = area under the curve; BART = Bayesian additive regression trees; HR = human reader; RF = random forest; ROC = receiver operating characteristic.

### Performance with limited features

The performance metric for each case of limited features is shown in Figure S3. The accuracy for the most important features varied by quantity, yielding scores of 0.79, 0.81, 0.81, and 0.77 for the respective amounts of 5, 10, 15, and all features. The top 15 features comprised a combination of the following, each with specific filters applied: GLSZM, GLCM, first-order statistics, and GLDM (see Figure S4).

## Discussion

In this work, we demonstrate that BART is capable of diagnosing ALT from MRI radiomics data as well as human readers. In addition, the results show that the model can be optimized with as few as 5 predictors, with 78% accuracy compared to 77% when all are included. The BART prediction could be developed to act as a second opinion to increase the confidence of the diagnosis, potentially reducing the need for a biopsy confirmation in some cases.

The ROC analysis from 10-fold cross-validation shows that the BART model has a 5.5% lower AUC than the human reader, indicating slightly better accuracy for the human reader, though not significantly different (*P* = .9957). The kappa score of 0.64 suggests good agreement.

External validation revealed that sensitivity varies with inter-cohort differences despite data harmonization, while BART's specificity generally improves, outperforming humans in cohorts from sites A and C but slightly decreasing in others. Overall accuracy across cohorts differs by up to 22%. Due to limited, harmonized data, these results are acceptable, but future work should address variability sources.

Relative to other ML models, such as gradient boosting machine (GBM), Least absolute shrinkage and selection operator (LASSO), neural nets, and RF, evidence shows that BART is more efficient and flexible.^15^ According to the literature, a validity study showed that BART was more sensitive in predicting colon cancer from microarray colon data than RF and GBM.^17^ In the work by Spaanderman et al, they reported an AUC for optimized traditional ML models of 0.74, which is much lower than BART or RF reported here.^14^ This could be explained by the smaller dataset used for training in comparison to this work (423 vs 150 subjects). BART has not been used in the texture analysis of MRI images before, and so BART represents a novel ML approach for ALT classification.

One of the limitations of this study was the vast variability of cohort size. This might contribute to a bias in the training of the model toward data from larger cohorts; however, a site imbalance evaluation showed that there were no major biases because of it. We conducted subsampling analyses to balance cohort prevalence, which demonstrated consistent performance metrics with the full dataset, confirming that results are not influenced by Cohort A's larger sample size (Table S5). We evaluated model performance with and without ComBat harmonization, finding that it reduces site-related variance while preserving predictive signal, as shown by stable performance across sites. Notably, harmonization increased specificity but decreased sensitivity in external validation. We intend to investigate this in future work.

The effect might be further reduced with more advanced harmonization techniques—for example, a more recent ComBat version is available—and by curating cohorts with data of equal sample sizes and disease prevalence. The image segmentation issue must also be addressed with automated tools to ease clinical translation. For example, Spaanderman et al demonstrated a deep learning tool to segment lipoma images for radiomics analysis.

Another limitation is that only a single human reader was included, and the inter-reader variability was not evaluated. This factor may impact the results shown in this work, possibly making the ML outperform the human reader.

In this study, the large overall cohort and multi-institutional data provide a robust basis for the accurate evaluation of BART. Our future work will involve understanding the effects of cohort variability and how to reduce the effects.

BART’s clinical service may include acting as a second reader, providing case-level risk assessments with quantified uncertainty to aid review prioritization, biopsy support, and multidisciplinary planning. Further investigation is needed, as the interaction between BART and human readers and how this would impact reader confidence or subsequent decision-making was not addressed in the current work. Patients with a high clinical and radiological suspicion of lipoma, confirmed by radiomics, might avoid diagnostic biopsy. Surgery could be reserved for symptom development or tumor changes on follow-up. The model’s probabilistic outputs provide interpretability and consistency, addressing radiologic challenges. With external validation and integration into picture archiving and communication system (PACS) or electronic health records (EHR), BART may improve diagnostic efficiency, reduce variability, and support limited subspecialty settings. The feature extraction process might be simplified to reduce the computation burden by targeting a smaller number of radiomic features. The results show that with as few as 15 features, the model could perform as well as an experienced radiologist. The most important features were those that were based on gray-level uniformity and anatomical edge distinction. Our variable importance analysis revealed 5 radiomic domains that differentiate ALT from lipoma. Heterogeneity/granularity features (eg, Dependence Entropy, Gray Level Non-Uniformity) were higher in ALTs, reflecting irregular septa and nodules, while lipomas appeared more uniform. Zone organization measures showed ALTs often had large bright regions, whereas lipomas displayed broad dark zones of suppressed fat. Edge conspicuity (GLCM contrast, Cluster Shade) and histogram tails highlighted sharper transitions and bright foci in ALTs vs smoother, darker profiles in lipomas. Morphology (size, elongation) was supported but did not drive discrimination. Together, these domains align with known imaging hallmarks of ALT vs lipoma (see Supplementary Information—Limited feature analysis).

The characteristics of images may help radiologists identify potential BART failures. Patterns where both BART and human reader failed similarly or contradicted each other were examined (Figures S2 and S3). False positives are often linked to deep lesions and large sizes, common in lipomas. Lesions in the proximal lower extremity may be misdiagnosed as ALT rather than lipoma, as this area frequently hosts ALTs. Features like thick septa, nodularity, and complex architecture are seen in ALTs but are not exclusive to them, also appearing in lipomas. These features assist in correct diagnoses but can also lead to false positives. Conversely, the absence of these features in ALTs can result in false negatives.

These results support further research on BART in future studies to assess its impact on diagnostic accuracy, workflow, and management. BART's calibrated risk estimates can aid decision-making and clinical integration, such as biopsy triage or surgery planning. These studies should also examine user trust and interpretability using BART's feature importance, ultimately evaluating if it improves patient outcomes, reduces variability, and broadens radiology use. An important milestone in this work is to develop a deployment monitoring strategy that will include performance monitoring, data drift detection, and model retraining if performance degrades.

## Conclusion

In this work, we demonstrated that the BART model of regression trees can be used to classify ALT from lipoma. This is the first time the BART model has been applied to this type of cancer classification, and we demonstrated that BART is comparable to the human observer for ALT diagnosis. BART should be further investigated with larger cohorts toward clinical translation.

## Supplementary Material

umaf036_Supplementary_Data
