# Supplementary material for: Bayesian additive regression trees for machine learning to classify benign vs atypical lipomatous tumors on MRI
Source: Radiol Adv. 2025 Oct 6;2(5):umaf036. doi: 10.1093/radadv/umaf036 (PMC12548370; doi:10.1093/radadv/umaf036)
Supplement: umaf036_Supplementary_Data [file umaf036_supplementary_data.zip › Supplementary information.pdf]

Supplementary information for Bayesian Additive Regression Trees machine learning to classify benign vs. atypical lipomatous tumors on MRI

A. Supplementary figures

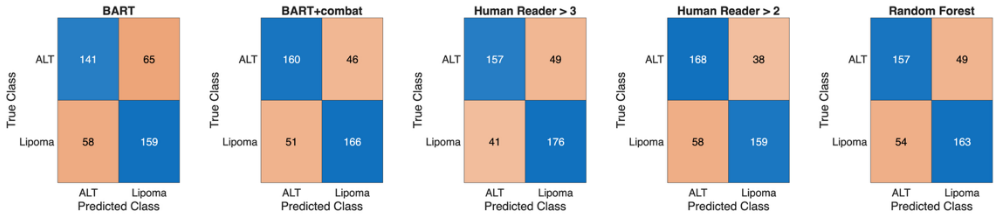

Figure S1 Confusion matrix from the BART model with and without ComBat harmonization and the human reader for a scale threshold of 2 and 3. An experienced musculoskeletal radiologist reviewed all the acquired MR series for each patient, and categorized the impression on a 5-point diagnostic score; where 1 = definitely lipoma, 2 = probably lipoma, 3 = equivocal, 4 = probably ALT, and 5 = definitely ALT.

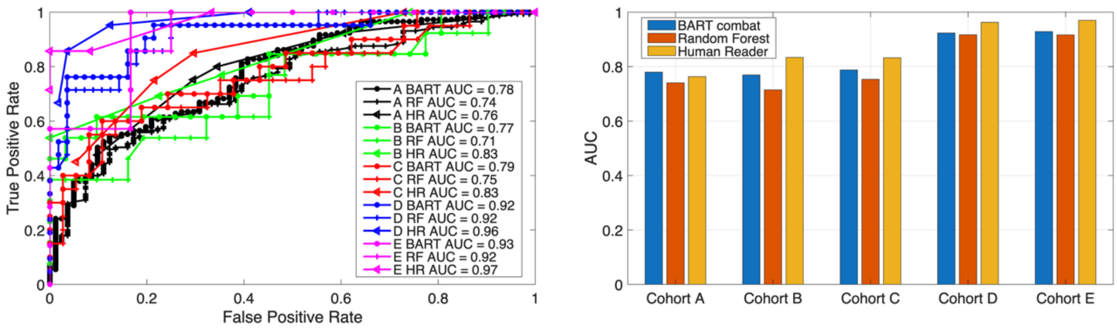

Figure S2 Classification performance for the BART model with external validation. One cohort is left out of training and used for testing. ROC curves on the left and AUC values is shown the right.

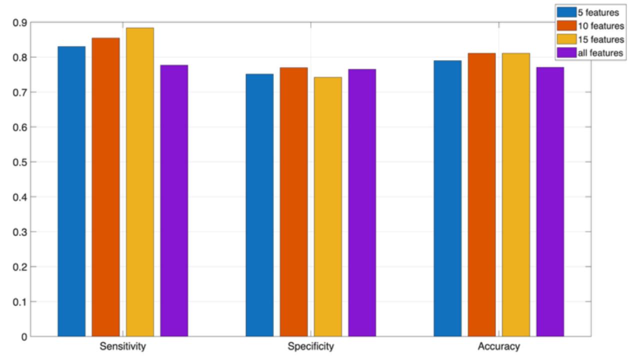

Figure S3 Classification performance for the BART model when the impact of features upon classification is considered, based on the variable inclusion proportion (VIP). The VIP is the proportion of times each predictor is chosen as a splitting rule divided by the total number of splitting rules appearing in the model, it provides a metric for the most important features. Models were trained with the 5, 10, 15 most important features.

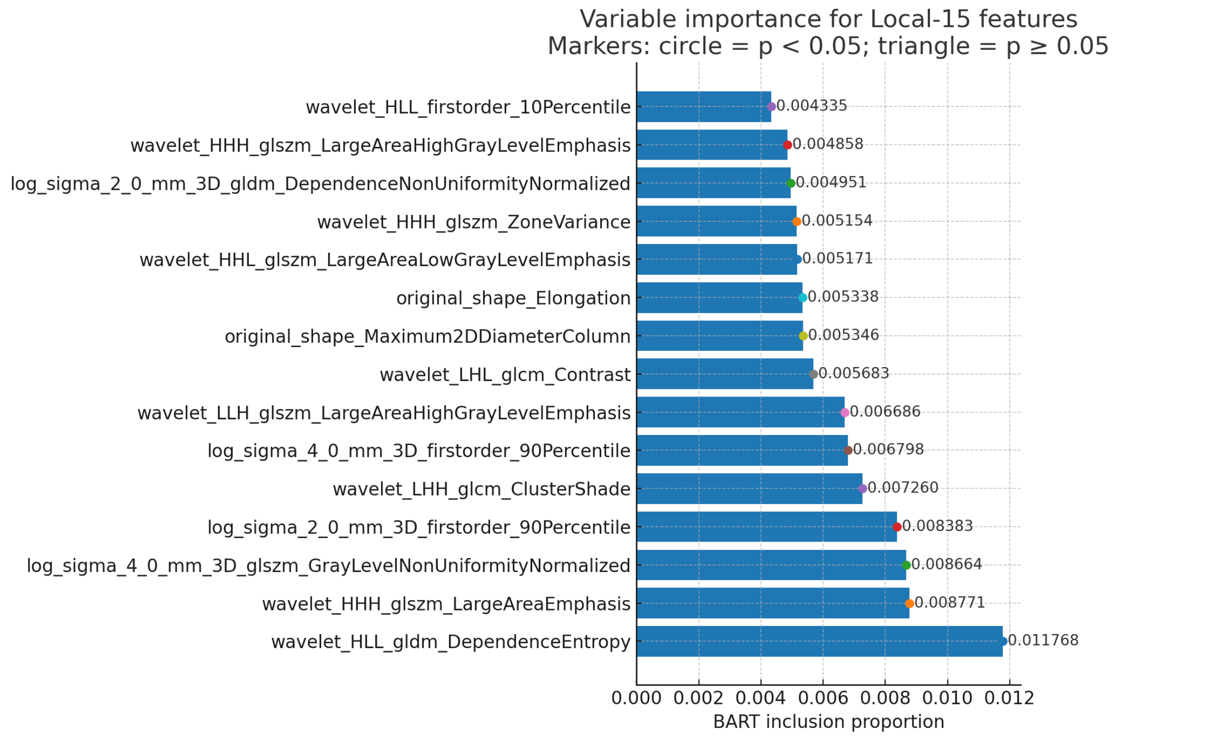

Figure S4 Bar graph of variable importance of 15 features.

B. Supplementary tables

Table S1 Scanners used to acquire the data	
Vendor	Model
Hitachi,	Airis II 0.7T
Toshiba	Titan
Siemens	Aera 1.5T, Avanto 1.5T, Espree 1.5T, Skyra 3.0T, Symphony 1.5T, Verio 3.0T, Trio Tim
Philips	Achieva 1.5T, Eclipse 1.5T, Gyroscan Intera 1.5, Ingenia 3.0T,
GE	S Discovery MR750 3.0T, MSK extreme, Optima MR450w 1.5T, Signa excite 1.5, Signa genesis 1.5T, Signa, HDe 1.5T, Signa HDx 3.0T, Signa HDxt 1.5T

Table S2 Pyradiomic features used in this study. The radiomic features included in this work covered the complete list available with the Pyradiomics libraries. The reader is encouraged to seek a full description and definitions on the Pyradiomics website, https://pyradiomics.readthedocs.io/en/latest/index.html.

Category	# Feature	What They Capture	Examples
**First Order**	19	Intensity distribution within ROI (histogram-based)	Mean, Median, Entropy, Skewness, Kurtosis
**Shape (2D)**	10	Morphology and geometric properties of ROI	Volume, Surface Area, Sphericity, Elongation

Table S2 Pyradiomic features used in this study. The radiomic features included in this work covered the complete list available with the Pyradiomics libraries. The reader is encouraged to seek a full description and definitions on the Pyradiomics website, https://pyradiomics.readthedocs.io/en/latest/index.html.			
**Category**	**# Feature**	**What They Capture**	**Examples**
**GLCM** (Gray Level Co-occurrence Matrix)	24	Spatial co-occurrence of gray levels (texture)	Contrast, Correlation, Homogeneity, Cluster Shade
**GLRLM** (Gray Level Run Length Matrix)	16	Length of consecutive runs of gray levels	Short Run Emphasis, Long Run Emphasis, Run Percentage
**GLSZM** (Gray Level Size Zone Matrix)	16	Size of homogeneous zones (connected voxels of same intensity)	Small Area Emphasis, Large Area Emphasis, Zone Entropy
**GLDM** (Gray Level Dependence Matrix)	14	Number of connected voxels depending on a center voxel	Dependence Non-Uniformity, Dependence Entropy
**NGTDM** (Neighborhood Gray Tone Difference Matrix)	5	Differences between a voxel and its neighbors	Coarseness, Contrast, Busyness, Complexity

Table S3 Feature importance and permutation P value			
Rank	Feature	Importance	Permutation p
1	wavelet_HLL_gldm_DependenceEntropy	0.011768	0.010
2	wavelet_HHH_glszm_LargeAreaEmphasis	0.008771	0.020
3	log_sigma_4_0_mm_3D_glszm_GrayLevelNonUniformityNormalized	0.008664	0.000
4	log_sigma_2_0_mm_3D_firstorder_90Percentile	0.008383	0.000
5	wavelet_LHH_glcm_ClusterShade	0.007260	0.000
6	log_sigma_4_0_mm_3D_firstorder_90Percentile	0.006798	0.000
7	wavelet_LLH_glszm_LargeAreaHighGrayLevelEmphasis	0.006686	0.000
8	wavelet_LHL_glcm_Contrast	0.005683	0.020
9	original_shape_Maximum2DDiameterColumn	0.005346	0.010
10	original_shape_Elongation	0.005338	0.000
11	wavelet_HHL_glszm_LargeAreaLowGrayLevelEmphasis	0.005171	0.000
12	wavelet_HHH_glszm_ZoneVariance	0.005154	0.000
13	log_sigma_2_0_mm_3D_gldm_DependenceNonUniformityNormalized	0.004951	0.010
14	wavelet_HHH_glszm_LargeAreaHighGrayLevelEmphasis	0.004858	0.000
15	wavelet_HLL_firstorder_10Percentile	0.004335	0.000

Table S4 Definitions and descriptions of features.

#	Feature	Filter (pre-proc)	Family	Short definition	Interpretation (higher →)
1	wavelet_HLL_gldm_DependenceEntropy	Wavelet HLL	GLDM	Shannon entropy of the GLDM probability distribution over dependence counts.	Greater textural randomness/heterogeneity.
2	wavelet_HHH_glszm_LargeAreaEmphasis	Wavelet HHH	GLSZM	Emphasis on large homogeneous zones (weights zone size).	Coarser texture; large uniform regions.
3	log_sigma_4_0_mm_3D_glszm_GrayLevelNonUniformityNormalized	LoG σ=4 mm	GLSZM	Normalized variability of gray levels across zones.	More gray-level variability across zones (lower = more uniform).
4	log_sigma_2_0_mm_3D_firstorder_90Percentile	LoG σ=2 mm	First-order	90th percentile of voxel intensities.	Brighter high-end tail of the intensity distribution.
5	wavelet_LHH_glcm_ClusterShade	Wavelet LHH	GLCM	Third central moment of the GLCM; measures distribution asymmetry.	More asymmetric/“skewed” co-occurrence patterns.
6	log_sigma_4_0_mm_3D_firstorder_90Percentile	LoG σ=4 mm	First-order	90th percentile after stronger smoothing (coarser scale).	Brighter high-end tail at a coarser spatial scale.
7	wavelet_LLH_glszm_LargeAreaHighGrayLevelEmphasis	Wavelet LLH	GLSZM	Emphasis on large zones with high gray levels.	Large bright homogeneous areas.
8	wavelet_LHL_glcm_Contrast	Wavelet LHL	GLCM	Weighted sum of squared gray-level differences (i−j)2p(i,j)(i-j)^2 p(i,j).	Higher local intensity variation/edge content.
9	original_shape_Maximum2DDiameterColumn	—	Shape	Largest pairwise 2D Euclidean distance in the plane orthogonal to the column axis.	Larger in-plane size/extent.
10	original_shape_Elongation	—	Shape	λminor/λmajor\sqrt{\lambda_{\text{minor}}/\lambda_{\text{major}}} from the ROI’s principal axes.	Closer to 1 = rounder; smaller = more elongated.
11	wavelet_HHL_glszm_LargeAreaLowGrayLevelEmphasis	Wavelet HHL	GLSZM	Emphasis on large zones with low gray levels.	Large dark homogeneous areas.
12	wavelet_HHH_glszm_ZoneVariance	Wavelet HHH	GLSZM	Variance of zone sizes in the GLSZM.	Greater variability in homogeneous-zone sizes.
13	log_sigma_2_0_mm_3D_gldm_DependenceNonUniformityNormalized	LoG σ=2 mm	GLDM	Normalized variability of dependence counts across the ROI.	More non-uniform dependence structure (lower = more uniform).
14	wavelet_HHH_glszm_LargeAreaHighGrayLevelEmphasis	Wavelet HHH	GLSZM	Emphasis on large zones with high gray levels.	Large bright homogeneous areas.

Table S4 Definitions and descriptions of features.					
#	Feature	Filter (pre-proc)	Family	Short definition	Interpretation (higher →)
15	wavelet_HLL_firstorder_10Percentile	Wavelet HLL	First-order	10th percentile of voxel intensities.	Darker low-end tail (more low-intensity voxels).

Table S5 Imbalance sensitivity analysis				
Cohort	Balance Sensitivity/Specificity		Imbalance	
	With ComBat	Without ComBat	With ComBat	Without ComBat
A	0.29/0.95	0.42/0.92	0.37/0.93	0.42/0.92
B	0.61/0.70	0.76/0.58	0.61/0.70	0.77/0.58
C	0.60/0.67	0.75/0.67	0.60/0.78	0.75/0.67
D	0.85/0.84	0.85/0.78	0.85/0.83	0.85/0.78
E	0.57/0.83	0.85/0.83	0.57/0.83	0.85/0.83

C. Supplementary methods

Bayesian additive regression tree model

BART is a machine learning method that models a complex relationship between inputs and an outcome by adding together the predictions from many small decision trees; it combines the flexibility of machine learning algorithms with the formality of likelihood-based inference. Each tree contributes only a small part to the final prediction, so the model builds up a flexible and accurate estimate in an additive way. Each tree is constrained by a regularization prior to being a weak learner, which restricts the depth of

the tree, so no single tree in the sum is overfitting the radiomic features. What makes BART special is that it uses Bayesian statistics to control the complexity of the model, automatically balancing fit and generalization. It does this by placing priors that prefer smaller trees and using a sampling algorithm (MCMC), iterative Bayesian backfitting from a posterior distribution of the parameters of the tree given the image features, to explore many possible combinations of trees.

In BART, a prior encodes our expert beliefs about the likely structure and strength of predictions before observing any radiomic features, guiding the model toward simpler, more plausible tree ensembles. The priors in BART serve to moderate the model's confidence: by constraining each tree to be a weak learner, they prevent overfitting and ensure that uncertainty in the model, reflected in the spread of posterior predictions, is faithfully represented. The result is a model that not only makes accurate predictions but also provides a natural measure of uncertainty, like confidence intervals, around those predictions. For a detailed and intuitive description of the BART model, the reader is directed to (16).

The BART model used in this work is represented by

$$y = \sum_{j=1}^{m} g\left(x; T_j, M_j\right) + \varepsilon, \quad (1)$$

where y denotes the predicted outcome for each patient, ALT or Lipoma, based on the set of radiomic features x extracted from each patient's MRI scans. The function $g\left(x; T_j, M_j\right)$ corresponds to the output of the jth tree in the ensemble, T_j, which can be seen as a vector decision rules guiding decision-making at each internal node – these take the form $\{x_q < c\}$, implying decisions are contingent on whether the radiomic feature x_q is

below a certain threshold c. The vector M_j contains the parameters that define the output for the terminal nodes (or leaves) of that tree. The model is completed by an additive gaussian error, $\varepsilon \sim N(0, \sigma^2)$. For comparison the BART model was matched against a standard random trees (RF) model using 500 trees.

Balanced Sampling Sensitivity

We performed repeated subsampling analyses to balance all cohorts prevalence. The resulting models showed performance metrics that were consistent with those obtained from the full dataset, indicating that our findings are not solely driven by the larger sample size at Cohort A. These results are now included in the discussion section and in the Supplementary information in Table S1.

Limited feature analysis

To identify the **most important** predictors, our objective was to **prioritize features that the model repeatedly uses**, beyond what would occur if the outcome carried no structure. We fit BART models and summarized each predictor's **importance** by its **variable inclusion proportion** (VIP), i.e., the average fraction of tree splits that used that predictor across trees and posterior draws. Because tree ensembles can assign small, spurious importance to many variables, we then constructed a **permutation null** by repeatedly shuffling the outcome, refitting the model, and recomputing inclusion proportions. This generates, for each variable, the distribution of importance one would expect if the outcome carried no signal.

More specifically, we report, for each predictor, a **permutation-calibrated p-value** that places its BART variable-importance in the context of a **no-signal** benchmark. Concretely, we compute the variable's inclusion proportion on the real labels and then recompute it across many **outcome permutations**; the p-value is the **fraction of permuted runs whose inclusion proportion is at least as large as the observed one**. Small values therefore indicate that the model's reliance on that feature is **unlikely to arise from chance**, given the same preprocessing, feature set, and learning algorithm. These p-values are **model- and pipeline-conditional** measures of *predictive contribution* (not causal effects), and they complement the raw inclusion proportions by calibrating them against the background importance that tree ensembles can assign even when no association is present.

Because selection used a **variable-specific (Local) permutation cutoff**, that is, features are considered only if their observed importance exceeds the 95th percentile of their own null: the selected features, will typically have **$p < 0.05$** (up to Monte-Carlo error). In our table, they range from **0.00 to 0.02**; zeros reflect values below the minimum attainable resolution $ 1/B$ given B permutations and rounding. We present these p-values **descriptively** to document robustness of feature importance; the final subset size and operating point were chosen based on **cross-validated predictive performance** (where the 15-feature model provided the best balance of sensitivity and specificity), not on p-values alone.

The observed importance of each variable can be compared to these null benchmarks using three complementary **thresholding rules**. The **Local rule** sets a variable-specific cutoff (we used the 95th percentile of that variable's own permutation distribution) and

keeps a predictor when its observed importance exceeds its **own** null. The **Global-SE rule** uses a single, more conservative cutoff based on the permutation mean plus a multiple of its standard deviation pooled across variables. The **Global-Max rule** is most stringent: it takes, across permutations, the distribution of the **maximum** importance seen among all variables and keeps only predictors that exceed the 95th percentile of that extreme-value distribution.

The Local rule may be preferable if the goal is prediction. It compares each feature's importance to **its own** permutation null. Texture features from different filters/scales have different baseline propensities to be used by trees; a single global cutoff treats them as exchangeable and often prunes moderate, truly helpful signals. Local preserves these **multiple, complementary cues** (fine/coarse heterogeneity, zone organization, edge irregularity, histogram tails, shape) that radiologists may also use.

Findings across rules

- **Local** we analyzed the predictive performance of multiple subsets of selected variables, and considered the **15 most included features** as the best option (more on this below). All had permutation benchmarks $p < 0.05$.
- **Global-SE** returned **4** features; importantly, all four are **contained within** the Local-15, indicating that Local extends a conservative core with additional, but still **non-null**, signals (e.g., fine vs coarse heterogeneity, zone organization, edge irregularity, histogram tails, shape).
- **Global-Max** returned **no features** in our setting, consistent with its stringency and the moderate effect sizes typical of radiomics.

In summary, the four features that pass the stricter Global-SE cutoff are **all contained** in the Local-15. In other words, Local includes the conservative core **plus** validated, smaller effects that boost predictive balance.

The table S4 includes the set of 15 selected features with corresponding importance weights and permutation p-values. Table S5 tabulates the meanings and definitions.

The model's most influential signals quantify **multi-scale heterogeneity**—the degree to which uniform fat is interrupted by thicker septa, stranding, or nodular non-fat tissue. Metrics such as GLDM **dependence entropy** and **dependence non-uniformity** (wavelet_HLL_gldm_DependenceEntropy; log_sigma_2_0_mm_3D_gldm_DependenceNonUniformityNormalized) rise as the internal texture becomes less predictable and more granular. At a broader spatial support, GLSZM **gray-level non-uniformity (normalized)** and **zone-size variance** (log_sigma_4_0_mm_3D_glszm_GrayLevelNonUniformityNormalized; wavelet_HHH_glszm_ZoneVariance) summarize the mixture of small and large homogeneous patches that typifies ALT when non-adipose components and irregular septa coexist with fat. Lipomas, in contrast, tend to keep these measures low because their signal is dominated by homogeneous fat with only delicate septa.

A second domain captures **how signal consolidates into large uniform "plates."** GLSZM **LargeAreaEmphasis** and the **high/low gray-level emphases** (wavelet_HHH_glszm_LargeAreaEmphasis; wavelet_LLH_glszm_LargeAreaHighGrayLevelEmphasis; wavelet_HHH_glszm_LargeAreaHighGrayLevelEmphasis; wavelet_HHL_glszm_LargeAreaLowGrayLevelEmphasis) tell us whether big contiguous

regions are predominantly bright or dark after filtering. On **fat-suppressed post-contrast** images, **bright** large-area emphases correspond to **enhancing septa or nodules** and therefore tend to be higher in ALT, whereas **large dark** emphases reflect **suppressed fat** and favor lipoma. The direction reverses on **non–fat-suppressed T1**, where fat is bright and often produces large bright plates in lipoma.

A third domain reflects **edge conspicuity and directional irregularity**—the visual impression of thick, uneven internal interfaces. GLCM **contrast** (wavelet_LHL_glcm_Contrast) quantifies sharp intensity transitions and typically increases with thicker, more conspicuous septa; GLCM **cluster shade** (wavelet_LHH_glcm_ClusterShade) tracks asymmetry in texture patterns and rises when stranding and nodularity break symmetry. Both measures are generally higher in ALT than in lipoma.

The **histogram tails** provide complementary, sequence-dependent cues about the burden of very bright or very dark voxels, stabilized at specific spatial scales. The **90th percentiles** at σ≈2 mm and σ≈4 mm (log_sigma_2_0_mm_3D_firstorder_90Percentile; log_sigma_4_0_mm_3D_firstorder_90Percentile) capture the bright tail: on **T1 non-FS**, this tail is driven by fat and thus tends to be higher in lipoma; on **fat-sat post-contrast**, it reflects enhancement and tends to be higher in ALT. The **10th percentile** (wavelet_HLL_firstorder_10Percentile) summarizes the dark tail: on fat-sat sequences, more dark voxels indicate larger volumes of suppressed fat (lipoma), whereas on non-FS T1, darker regions correspond to non-fat tissue and may favor ALT.

Finally, **morphology** contributes supportive context. **Maximum2DDiameterColumn** and **Elongation** (original_shape_Maximum2DDiameterColumn;

original_shape_Elongation) encode in-plane size and compactness. Although not decisive alone, these features align with clinical experience—ALTs often present larger or deeper—while acknowledging that elongation can vary with compartment and surgical plane.

Taken together, these 15 features operationalize what radiologists already use at the workstation: **heterogeneity and granularity** increase with ALT; **large bright plates** on fat-sat imply **enhancing tissue** (ALT) while **large dark plates** imply **suppressed fat** (lipoma); **edges and asymmetric texture** are more conspicuous in ALT; and **histogram tails** flip direction with sequence, favoring lipoma on T1 non-FS and ALT on post-contrast fat-sat. This blend of multi-scale texture, zone organization, intensity tails, and basic morphology explains why the 15-feature set yields a balanced improvement over smaller subsets that omit one or more of these domains.
